# 4-Hy­droxy-3-[(4-hy­droxy-6,7-dimethyl-2-oxo-2*H*-chromen-3-yl)(4-oxo-4*H*-chromen-3-yl)meth­yl]-6,7-dimethyl-2*H*-chromen-2-one

**DOI:** 10.1107/S1600536810041231

**Published:** 2010-10-20

**Authors:** Mohammad Asad, Chuan-Wei Oo, Hasnah Osman, Chin Sing Yeap, Hoong-Kun Fun

**Affiliations:** a School of Chemical Sciences, Universiti Sains Malaysia, 11800 USM, Penang, Malaysia; b X-ray Crystallography Unit, School of Physics, Universiti Sains Malaysia, 11800 USM, Penang, Malaysia

## Abstract

In the title compound, C_32_H_24_O_8_, the mol­ecular structure is disordered over two positions with refined site occupancies of 0.8746 (10) and 0.1254 (10). The mean plane of the three chromeno rings make dihedral angles with each other of 65.12 (4), 62.91 (4) and 59.70 (4)° in the major occupancy component and 59.1 (3), 66.1 (3) and 58.8 (3)° in the minor component. Intra­molecular O—H⋯O hydrogen bonds stabil­ize the mol­ecular structure and the crystal structure is stabilized by weak C–H⋯π and π–π inter­actions [centroid–centroid distances 3.496 (6)–3.672 (7) Å].

## Related literature

For general background and the biological activity of chromone heterocycle derivatives, see: Waring (1979 ▶); Dewick (1994 ▶); Rich (1990 ▶); Masami *et al.* (2007 ▶); Khan *et al.* (2010 ▶); Nawrot-Modranka *et al.* (2006 ▶); Ellis *et al.* (1978 ▶); Raj *et al.* (2010 ▶). For the stability of the temperature controller used in the data collection, see: Cosier & Glazer (1986 ▶).
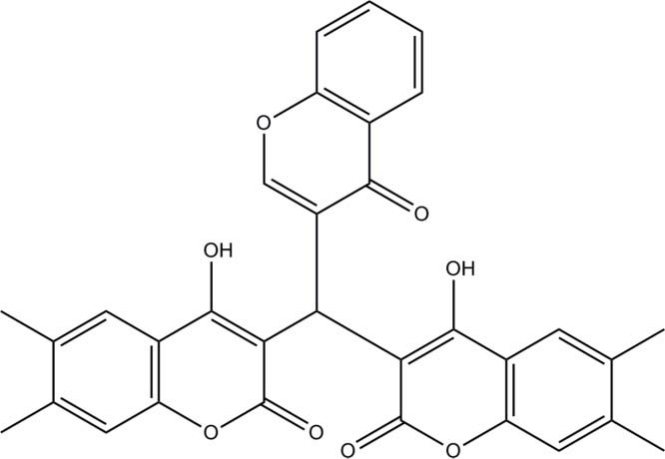


## Experimental

### 

#### Crystal data

C_32_H_24_O_8_
*M* *_r_* = 536.51
Monoclinic, 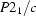

*a* = 13.6418 (12) Å
*b* = 10.5878 (9) Å
*c* = 21.2316 (15) Å
β = 123.480 (4)°
*V* = 2557.8 (4) Å^3^
*Z* = 4
Mo *K*α radiation
μ = 0.10 mm^−1^
*T* = 100 K
0.56 × 0.30 × 0.28 mm

#### Data collection

Bruker APEXII DUO CCD area-detector diffractometer
Absorption correction: multi-scan (*SADABS*; Bruker, 2009 ▶) *T*_min_ = 0.946, *T*_max_ = 0.973
37636 measured reflections
10128 independent reflections
7464 reflections with *I* > 2σ(*I*)
*R*_int_ = 0.037

#### Refinement

*R*[*F* ^2^ > 2σ(*F* ^2^)] = 0.051
*wR*(*F* ^2^) = 0.141
*S* = 1.03
10128 reflections
524 parameters
1102 restraints
H-atom parameters constrained
Δρ_max_ = 0.37 e Å^−3^
Δρ_min_ = −0.22 e Å^−3^

### 

Data collection: *APEX2* (Bruker, 2009 ▶); cell refinement: *SAINT* (Bruker, 2009 ▶); data reduction: *SAINT*; program(s) used to solve structure: *SHELXTL* (Sheldrick, 2008 ▶); program(s) used to refine structure: *SHELXTL*; molecular graphics: *SHELXTL*; software used to prepare material for publication: *SHELXTL* and *PLATON* (Spek, 2009 ▶).

## Supplementary Material

Crystal structure: contains datablocks global, I. DOI: 10.1107/S1600536810041231/rz2499sup1.cif

Structure factors: contains datablocks I. DOI: 10.1107/S1600536810041231/rz2499Isup2.hkl

Additional supplementary materials:  crystallographic information; 3D view; checkCIF report

## Figures and Tables

**Table 1:** Hydrogen-bond geometry (Å, °) *Cg*1, *Cg*2 and *Cg*3 are the centroids of the C13–C18, C2–C7 and C2*A*–C7*A* benzene rings, respectively.

*D*—H⋯*A*	*D*—H	H⋯*A*	*D*⋯*A*	*D*—H⋯*A*
O5—H5*A*⋯O7	0.82	1.83	2.6464 (19)	174
O6—H6*B*⋯O4	0.82	1.80	2.6141 (18)	172
C3—H3*A*⋯*Cg*1^i^	0.93	2.91	3.7691 (16)	155
C32—H32*B*⋯*Cg*2^ii^	0.96	2.70	3.499 (3)	140
C29*A*—H29*D*⋯*Cg*3^iii^	0.96	2.91	3.591 (15)	128

Symmetry codes: (i) 
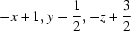
; (ii) 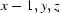; (iii) 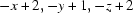.

**Fig. 1.**
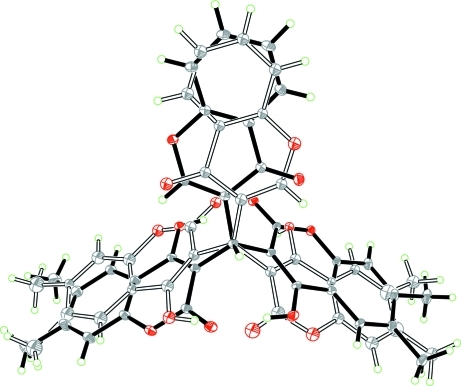
The molecular structure of the title compound, showing major (solid-bond) and minor (open-bond) disorder components.

**Fig. 2.**
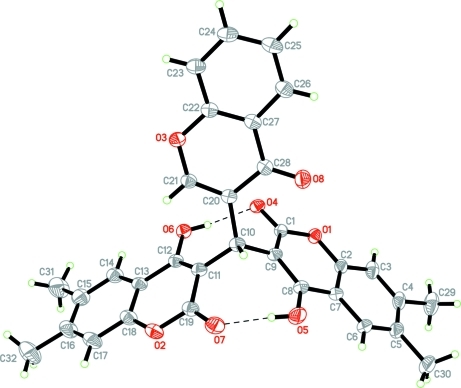
The major component of molecular structure of the title compound with atom labels and 50% probability ellipsoids for non-H atoms. Intramolecular hydrogen bonds are shown as dashed lines.

**Fig. 3.**
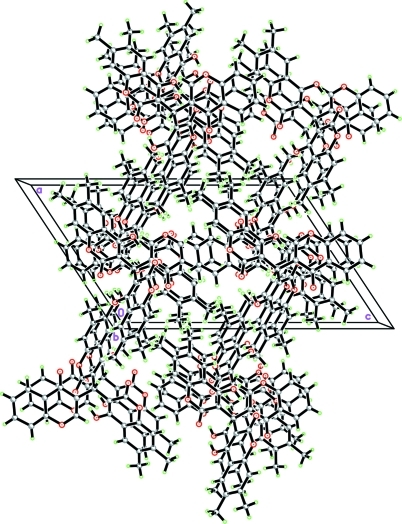
The crystal packing of the title compound viewed down the b axis. Only the major component of disorder is shown.

## supplementary crystallographic information

### Comment

The oxygen-containing chromone heterocycles are ubiquitous in plants (Waring, 1979). They form basic nucleus of flavones (Dewick, 1994) and have been found in many biologically active molecules with a broad range of applications in the pharmaceutical industry (Rich, 1990). These derivatives are an important class of compounds, possessing a large number of pharmacological activities such as anti-*HIV* (Masami *et al.*, 2007), potentially anti-inflammatory (Khan *et al.*, 2010), antibacterial (Nawrot-Modranka *et al.*, 2006), antiallergic (Ellis *et al.*, 1978) and anticancer (Raj *et al.*, 2010) activities.

The molecular structure of the title compound is disordered over two positions (Fig. 1) with refined site occupancies of 0.8746 (10) and 0.1254 (10). The mean plane of the three chromeno rings make dihedral angles of 65.12 (4), 62.91 (4) and 59.70 (4)° for major component and 59.1 (3), 66.1 (3) and 58.8 (3)° for minor component with each other. Intramolecular O5—H5A···O7 and O6—H6B···O4 hydrogen bonds (Table 1) stabilize the molecular structure and generate *S*(8) ring motifs (Fig. 2). The molecules are stacked down the *b* axis (Fig. 3) and stabilized by weak C–H···π and π···π interactions [*Cg*4···*Cg*5^iv^ of 3.496 (6) Å and *Cg*5···*Cg*6^v^ of 3.672 (7) Å; (iv) 1 - *x*, -1/2 + *y*, 3/2 - *z*; (v) 1 - *x*, 1/2 + *y*, 3/2 - *z. Cg*4, *Cg*5 and *Cg*6 are centroids of O2A–C18A–C13A–C12A–C11A–C19A, O3A–C21A–C20A–C28A–C27A–C22A and C13A–C18A ring, respectively].

### Experimental

A mixture of 6,7-dimethyl-4-hydroxycoumarin (1.05 mmol, 200 mg) and 3-formyl chromone (0.52 mmol, 91 mg) was prepared in ethanol (10 ml) and acetic acid (0.5 ml) was added to it. The reaction mixture was refluxed on water bath for 6 h. The completion of the reaction was monitored by TLC. After completion of the reaction, the crystalline solid was filtered, washed with ethanol and purified by recrystallization from chloroform-methanol mixture to give the pure title compound in 75% yield.

### Refinement

The molecular structure is disordered over two positions with refined site occupancies of 0.8746 (10) and 0.1254 (10). The minor component was refined isotropically. The same *U*_ij_ parameters were used for the atoms C5A/C31A/C32A. All disordered atoms were subjected to rigid bond and similarity restraints. One of the chromeno ring was restrained to be planar. H10A hydrogen atom was located in a difference Fourier map and refined using a riding model. The rest of hydrogen atoms were positioned geometrically [O–H = 0.82 Å; C–H = 0.93–0.96 Å] and refined using a riding model [*U*_iso_(H) = 1.5*U*_eq_(O); *U*_iso_(H) = 1.2–1.5*U*_eq_(C)]. A rotating-group model was applied for methyl groups.

### Crystal data

**Table:**

C_32_H_24_O_8_	*F*(000) = 1120
*M**_r_* = 536.51	*D*_x_ = 1.393 Mg m^−^^3^
Monoclinic, *P*2_1_/*c*	Mo *K*α radiation, λ = 0.71073 Å
Hall symbol: -P 2ybc	Cell parameters from 9464 reflections
*a* = 13.6418 (12) Å	θ = 3.2–33.7°
*b* = 10.5878 (9) Å	µ = 0.10 mm^−^^1^
*c* = 21.2316 (15) Å	*T* = 100 K
β = 123.480 (4)°	Block, colourless
*V* = 2557.8 (4) Å^3^	0.56 × 0.30 × 0.28 mm
*Z* = 4	

### Data collection

**Table:**

Bruker APEXII DUO CCD area-detector diffractometer	10128 independent reflections
Radiation source: fine-focus sealed tube	7464 reflections with *I* > 2σ(*I*)
graphite	*R*_int_ = 0.037
φ and ω scans	θ_max_ = 33.8°, θ_min_ = 2.2°
Absorption correction: multi-scan (*SADABS*; Bruker, 2009)	*h* = −21→21
*T*_min_ = 0.946, *T*_max_ = 0.973	*k* = −12→16
37636 measured reflections	*l* = −33→31

### Refinement

**Table:**

Refinement on *F*^2^	Primary atom site location: structure-invariant direct methods
Least-squares matrix: full	Secondary atom site location: difference Fourier map
*R*[*F*^2^ > 2σ(*F*^2^)] = 0.051	Hydrogen site location: inferred from neighbouring sites
*wR*(*F*^2^) = 0.141	H-atom parameters constrained
*S* = 1.03	*w* = 1/[σ^2^(*F*_o_^2^) + (0.0593*P*)^2^ + 0.7344*P*] where *P* = (*F*_o_^2^ + 2*F*_c_^2^)/3
10128 reflections	(Δ/σ)_max_ = 0.001
524 parameters	Δρ_max_ = 0.37 e Å^−^^3^
1102 restraints	Δρ_min_ = −0.22 e Å^−^^3^

### Special details

**Table:**

Experimental. The crystal was placed in the cold stream of an Oxford Cryosystems Cobra open-flow nitrogen cryostat (Cosier & Glazer, 1986) operating at 100.0 (1) K.
Geometry. All e.s.d.'s (except the e.s.d. in the dihedral angle between two l.s. planes) are estimated using the full covariance matrix. The cell e.s.d.'s are taken into account individually in the estimation of e.s.d.'s in distances, angles and torsion angles; correlations between e.s.d.'s in cell parameters are only used when they are defined by crystal symmetry. An approximate (isotropic) treatment of cell e.s.d.'s is used for estimating e.s.d.'s involving l.s. planes.
Refinement. Refinement of *F*^2^ against ALL reflections. The weighted *R*-factor *wR* and goodness of fit *S* are based on *F*^2^, conventional *R*-factors *R* are based on *F*, with *F* set to zero for negative *F*^2^. The threshold expression of *F*^2^ > σ(*F*^2^) is used only for calculating *R*-factors(gt) *etc*. and is not relevant to the choice of reflections for refinement. *R*-factors based on *F*^2^ are statistically about twice as large as those based on *F*, and *R*- factors based on ALL data will be even larger.

### Fractional atomic coordinates and isotropic or equivalent isotropic displacement parameters (Å^2^)

**Table:**

	*x*	*y*	*z*	*U*_iso_*/*U*_eq_	Occ. (<1)
C10	0.59934 (8)	0.98504 (9)	0.78627 (5)	0.02251 (17)	
H10A	0.6445	1.0562	0.8167	0.027*	
O1	0.73539 (7)	0.68760 (8)	0.76785 (4)	0.02341 (16)	0.8746 (10)
O2	0.47327 (9)	1.01052 (8)	0.90437 (5)	0.02818 (18)	0.8746 (10)
O3	0.37210 (7)	1.12802 (8)	0.59633 (5)	0.02604 (17)	0.8746 (10)
O4	0.55778 (7)	0.76495 (7)	0.69426 (4)	0.02260 (15)	0.8746 (10)
O5	0.82100 (10)	0.95360 (11)	0.93221 (5)	0.0371 (2)	0.8746 (10)
H5A	0.7628	0.9865	0.9274	0.056*	0.8746 (10)
O6	0.38782 (10)	0.81531 (9)	0.71296 (5)	0.02352 (17)	0.8746 (10)
H6B	0.4453	0.8032	0.7108	0.035*	0.8746 (10)
O7	0.64315 (8)	1.06877 (9)	0.92532 (5)	0.03188 (19)	0.8746 (10)
O8	0.71448 (9)	1.01343 (10)	0.70554 (6)	0.0333 (2)	0.8746 (10)
C1	0.65408 (11)	0.77741 (12)	0.75365 (6)	0.0200 (2)	0.8746 (10)
C2	0.84098 (10)	0.68293 (11)	0.83692 (6)	0.0238 (2)	0.8746 (10)
C3	0.91643 (11)	0.58524 (12)	0.84766 (8)	0.0300 (2)	0.8746 (10)
H3A	0.8965	0.5289	0.8087	0.036*	0.8746 (10)
C4	1.02195 (12)	0.57262 (15)	0.91709 (10)	0.0328 (3)	0.8746 (10)
C5	1.05388 (13)	0.6590 (2)	0.97607 (9)	0.0355 (4)	0.8746 (10)
C6	0.97653 (11)	0.75649 (15)	0.96287 (7)	0.0334 (3)	0.8746 (10)
H6A	0.9967	0.8143	1.0012	0.040*	0.8746 (10)
C7	0.86887 (10)	0.76993 (14)	0.89335 (7)	0.0252 (2)	0.8746 (10)
C8	0.78733 (10)	0.87052 (12)	0.87659 (6)	0.0255 (2)	0.8746 (10)
C9	0.68462 (9)	0.87684 (10)	0.80711 (6)	0.02114 (19)	0.8746 (10)
C11	0.51591 (10)	0.96109 (10)	0.81050 (6)	0.02201 (19)	0.8746 (10)
C12	0.41935 (10)	0.88413 (10)	0.77386 (6)	0.02157 (19)	0.8746 (10)
C13	0.33957 (12)	0.87755 (13)	0.79841 (9)	0.0235 (2)	0.8746 (10)
C14	0.23512 (12)	0.80865 (12)	0.76169 (7)	0.0297 (2)	0.8746 (10)
H14A	0.2151	0.7601	0.7197	0.036*	0.8746 (10)
C15	0.16077 (16)	0.81163 (16)	0.78707 (10)	0.0369 (3)	0.8746 (10)
C16	0.19036 (18)	0.8865 (2)	0.84998 (13)	0.0388 (4)	0.8746 (10)
C17	0.29428 (13)	0.95199 (13)	0.88778 (8)	0.0351 (3)	0.8746 (10)
H17A	0.3148	0.9996	0.9302	0.042*	0.8746 (10)
C18	0.36863 (11)	0.94693 (11)	0.86242 (7)	0.0273 (2)	0.8746 (10)
C19	0.54895 (12)	1.01699 (11)	0.88165 (6)	0.0250 (2)	0.8746 (10)
C20	0.54055 (12)	1.03192 (10)	0.70468 (6)	0.02065 (19)	0.8746 (10)
C21	0.42855 (12)	1.07318 (11)	0.66505 (6)	0.0229 (2)	0.8746 (10)
H21A	0.3862	1.0629	0.6871	0.027*	0.8746 (10)
C22	0.43328 (11)	1.14560 (10)	0.56314 (7)	0.0232 (2)	0.8746 (10)
C23	0.37170 (12)	1.20468 (12)	0.49245 (7)	0.0284 (2)	0.8746 (10)
H23A	0.2943	1.2307	0.4705	0.034*	0.8746 (10)
C24	0.42912 (16)	1.22319 (12)	0.45629 (7)	0.0309 (3)	0.8746 (10)
H24A	0.3894	1.2612	0.4089	0.037*	0.8746 (10)
C25	0.54523 (15)	1.18597 (13)	0.48947 (8)	0.0331 (3)	0.8746 (10)
H25A	0.5828	1.1999	0.4645	0.040*	0.8746 (10)
C26	0.60504 (12)	1.12824 (12)	0.55951 (8)	0.0293 (2)	0.8746 (10)
H26A	0.6828	1.1035	0.5816	0.035*	0.8746 (10)
C27	0.54863 (12)	1.10686 (11)	0.59737 (7)	0.0231 (2)	0.8746 (10)
C28	0.61095 (10)	1.04642 (10)	0.67235 (6)	0.0235 (2)	0.8746 (10)
C29	1.10440 (15)	0.46606 (17)	0.92871 (12)	0.0492 (4)	0.8746 (10)
H29A	1.0713	0.4187	0.8827	0.074*	0.8746 (10)
H29B	1.1153	0.4115	0.9682	0.074*	0.8746 (10)
H29C	1.1789	0.5004	0.9425	0.074*	0.8746 (10)
C30	1.16883 (12)	0.6490 (2)	1.05083 (9)	0.0493 (4)	0.8746 (10)
H30A	1.1736	0.7141	1.0839	0.074*	0.8746 (10)
H30B	1.2321	0.6586	1.0438	0.074*	0.8746 (10)
H30C	1.1744	0.5678	1.0727	0.074*	0.8746 (10)
C31	0.05114 (17)	0.7292 (2)	0.74713 (11)	0.0545 (5)	0.8746 (10)
H31A	0.0434	0.6905	0.7037	0.082*	0.8746 (10)
H31B	−0.0168	0.7804	0.7314	0.082*	0.8746 (10)
H31C	0.0576	0.6649	0.7811	0.082*	0.8746 (10)
C32	0.1078 (2)	0.8923 (2)	0.87623 (14)	0.0566 (5)	0.8746 (10)
H32A	0.1389	0.9490	0.9184	0.085*	0.8746 (10)
H32B	0.0996	0.8095	0.8913	0.085*	0.8746 (10)
H32C	0.0323	0.9220	0.8358	0.085*	0.8746 (10)
O1A	0.8805 (6)	0.8897 (6)	0.9690 (4)	0.0415 (16)*	0.1254 (10)
O2A	0.3213 (6)	0.7840 (7)	0.7070 (4)	0.0353 (14)*	0.1254 (10)
O3A	0.6758 (6)	1.0511 (7)	0.6468 (4)	0.0364 (15)*	0.1254 (10)
O4A	0.7459 (7)	1.0309 (8)	0.9456 (5)	0.051 (2)*	0.1254 (10)
O5A	0.6824 (6)	0.7439 (7)	0.7564 (3)	0.0205 (14)*	0.1254 (10)
H5AA	0.6438	0.6855	0.7574	0.031*	0.1254 (10)
O6A	0.5112 (8)	1.0139 (7)	0.8896 (4)	0.0353 (17)*	0.1254 (10)
H6AB	0.5812	1.0161	0.9052	0.053*	0.1254 (10)
O7A	0.4578 (5)	0.7878 (6)	0.6821 (3)	0.0300 (13)*	0.1254 (10)
O8A	0.3923 (7)	1.1013 (8)	0.6664 (4)	0.0350 (17)*	0.1254 (10)
C1A	0.7792 (9)	0.9484 (9)	0.9212 (6)	0.041 (3)*	0.1254 (10)
C2A	0.9176 (7)	0.7844 (7)	0.9466 (5)	0.0329 (18)*	0.1254 (10)
C3A	1.0253 (8)	0.7332 (9)	1.0020 (6)	0.042 (2)*	0.1254 (10)
H3AA	1.0689	0.7675	1.0503	0.050*	0.1254 (10)
C4A	1.0660 (12)	0.6290 (12)	0.9831 (8)	0.060 (6)*	0.1254 (10)
C5A	1.0016 (12)	0.5766 (13)	0.9100 (9)	0.069 (3)*	0.1254 (10)
C6A	0.8923 (7)	0.6330 (8)	0.8564 (5)	0.0255 (15)*	0.1254 (10)
H6AA	0.8469	0.6001	0.8078	0.031*	0.1254 (10)
C7A	0.8538 (6)	0.7333 (7)	0.8751 (4)	0.0176 (16)*	0.1254 (10)
C8A	0.7432 (6)	0.7985 (7)	0.8234 (4)	0.0295 (16)*	0.1254 (10)
C9A	0.7094 (7)	0.8993 (7)	0.8435 (5)	0.0287 (16)*	0.1254 (10)
C11A	0.4883 (8)	0.9182 (9)	0.7822 (5)	0.0289 (16)*	0.1254 (10)
C12A	0.4555 (8)	0.9462 (10)	0.8290 (5)	0.0382 (19)*	0.1254 (10)
C13A	0.3474 (8)	0.9000 (10)	0.8161 (6)	0.023 (2)*	0.1254 (10)
C14A	0.2988 (10)	0.9292 (12)	0.8567 (7)	0.046 (2)*	0.1254 (10)
H14B	0.3382	0.9809	0.8991	0.055*	0.1254 (10)
C15A	0.1817 (14)	0.8754 (18)	0.8305 (9)	0.048 (5)*	0.1254 (10)
C16A	0.1241 (9)	0.7972 (10)	0.7680 (6)	0.027 (2)*	0.1254 (10)
C17A	0.1716 (10)	0.7720 (12)	0.7287 (7)	0.048 (2)*	0.1254 (10)
H17B	0.1318	0.7211	0.6860	0.058*	0.1254 (10)
C18A	0.2820 (9)	0.8227 (10)	0.7524 (6)	0.041 (2)*	0.1254 (10)
C19A	0.4243 (10)	0.8301 (12)	0.7212 (7)	0.035 (3)*	0.1254 (10)
C20A	0.5847 (8)	1.0206 (11)	0.7164 (6)	0.033 (2)*	0.1254 (10)
C21A	0.6731 (9)	1.0117 (10)	0.7060 (5)	0.0286 (19)*	0.1254 (10)
H21B	0.7413	0.9732	0.7448	0.034*	0.1254 (10)
C22A	0.5769 (8)	1.1114 (9)	0.5894 (5)	0.024 (2)*	0.1254 (10)
C23A	0.5778 (9)	1.1551 (10)	0.5272 (6)	0.0317 (19)*	0.1254 (10)
H23B	0.6432	1.1436	0.5247	0.038*	0.1254 (10)
C24A	0.4813 (10)	1.2138 (10)	0.4716 (6)	0.031 (2)*	0.1254 (10)
H24B	0.4797	1.2445	0.4300	0.037*	0.1254 (10)
C25A	0.3878 (9)	1.2287 (11)	0.4753 (6)	0.034 (2)*	0.1254 (10)
H25B	0.3217	1.2679	0.4352	0.041*	0.1254 (10)
C26A	0.3840 (8)	1.1899 (9)	0.5342 (5)	0.0336 (18)*	0.1254 (10)
H26B	0.3176	1.2041	0.5351	0.040*	0.1254 (10)
C27A	0.4813 (8)	1.1283 (8)	0.5935 (5)	0.0260 (16)*	0.1254 (10)
C28A	0.4785 (7)	1.0841 (8)	0.6611 (4)	0.0280 (16)*	0.1254 (10)
C29A	1.1849 (10)	0.5735 (12)	1.0420 (6)	0.045 (2)*	0.1254 (10)
H29D	1.1801	0.4830	1.0398	0.068*	0.1254 (10)
H29E	1.2075	0.6015	1.0913	0.068*	0.1254 (10)
H29F	1.2424	0.6009	1.0322	0.068*	0.1254 (10)
C30A	1.0507 (12)	0.4649 (12)	0.8946 (8)	0.049 (3)*	0.1254 (10)
H30D	0.9984	0.4397	0.8429	0.074*	0.1254 (10)
H30E	1.0595	0.3967	0.9271	0.074*	0.1254 (10)
H30F	1.1260	0.4859	0.9038	0.074*	0.1254 (10)
C31A	0.1392 (18)	0.918 (2)	0.8814 (12)	0.069 (3)*	0.1254 (10)
H31D	0.0589	0.9452	0.8504	0.103*	0.1254 (10)
H31E	0.1874	0.9862	0.9131	0.103*	0.1254 (10)
H31F	0.1450	0.8482	0.9123	0.103*	0.1254 (10)
C32A	0.0079 (13)	0.7464 (19)	0.7496 (11)	0.069 (3)*	0.1254 (10)
H32D	−0.0198	0.6832	0.7109	0.103*	0.1254 (10)
H32E	−0.0482	0.8140	0.7320	0.103*	0.1254 (10)
H32F	0.0177	0.7096	0.7941	0.103*	0.1254 (10)

### Atomic displacement parameters (Å^2^)

**Table:**

	*U*^11^	*U*^22^	*U*^33^	*U*^12^	*U*^13^	*U*^23^
C10	0.0265 (4)	0.0190 (4)	0.0206 (4)	−0.0010 (3)	0.0121 (3)	−0.0029 (3)
O1	0.0225 (3)	0.0216 (3)	0.0249 (4)	0.0024 (3)	0.0124 (3)	−0.0007 (3)
O2	0.0399 (5)	0.0251 (4)	0.0264 (4)	0.0006 (3)	0.0226 (4)	−0.0008 (3)
O3	0.0280 (4)	0.0259 (4)	0.0247 (4)	0.0035 (3)	0.0148 (3)	0.0055 (3)
O4	0.0241 (3)	0.0214 (3)	0.0183 (3)	−0.0005 (3)	0.0092 (3)	−0.0027 (3)
O5	0.0281 (5)	0.0508 (6)	0.0222 (4)	0.0020 (5)	0.0075 (4)	−0.0132 (4)
O6	0.0255 (5)	0.0213 (4)	0.0227 (4)	−0.0005 (4)	0.0127 (4)	−0.0021 (3)
O7	0.0363 (5)	0.0322 (4)	0.0256 (4)	−0.0021 (4)	0.0160 (4)	−0.0099 (3)
O8	0.0274 (5)	0.0360 (5)	0.0387 (5)	0.0038 (4)	0.0195 (4)	0.0097 (4)
C1	0.0222 (5)	0.0185 (5)	0.0200 (5)	0.0017 (4)	0.0120 (4)	0.0006 (4)
C2	0.0212 (5)	0.0253 (5)	0.0264 (5)	0.0021 (4)	0.0141 (4)	0.0057 (4)
C3	0.0252 (5)	0.0252 (5)	0.0420 (7)	0.0033 (4)	0.0200 (5)	0.0071 (5)
C4	0.0227 (5)	0.0330 (6)	0.0440 (7)	0.0055 (5)	0.0193 (5)	0.0176 (5)
C5	0.0220 (6)	0.0513 (9)	0.0322 (7)	0.0034 (6)	0.0143 (5)	0.0169 (7)
C6	0.0239 (5)	0.0516 (8)	0.0214 (5)	0.0034 (5)	0.0105 (4)	0.0078 (5)
C7	0.0230 (5)	0.0330 (6)	0.0187 (5)	0.0021 (5)	0.0109 (4)	0.0027 (5)
C8	0.0237 (5)	0.0328 (5)	0.0179 (4)	−0.0009 (4)	0.0100 (4)	−0.0028 (4)
C9	0.0226 (4)	0.0215 (4)	0.0166 (4)	−0.0008 (4)	0.0092 (4)	−0.0019 (3)
C11	0.0277 (5)	0.0185 (4)	0.0196 (4)	0.0019 (4)	0.0130 (4)	−0.0002 (4)
C12	0.0262 (5)	0.0176 (4)	0.0204 (4)	0.0027 (4)	0.0126 (4)	0.0024 (3)
C13	0.0315 (6)	0.0195 (5)	0.0228 (6)	0.0031 (4)	0.0170 (5)	0.0041 (5)
C14	0.0336 (6)	0.0278 (5)	0.0293 (5)	0.0000 (5)	0.0184 (5)	0.0055 (4)
C15	0.0343 (8)	0.0415 (8)	0.0367 (8)	−0.0054 (7)	0.0208 (7)	0.0075 (6)
C16	0.0487 (9)	0.0403 (9)	0.0430 (10)	0.0034 (6)	0.0350 (8)	0.0108 (7)
C17	0.0514 (8)	0.0311 (6)	0.0366 (6)	0.0029 (5)	0.0331 (6)	0.0051 (5)
C18	0.0370 (6)	0.0225 (5)	0.0282 (5)	0.0031 (4)	0.0216 (5)	0.0056 (4)
C19	0.0320 (6)	0.0211 (5)	0.0239 (5)	0.0022 (4)	0.0167 (5)	−0.0001 (4)
C20	0.0261 (6)	0.0162 (4)	0.0207 (5)	−0.0018 (4)	0.0135 (4)	−0.0015 (3)
C21	0.0278 (5)	0.0199 (4)	0.0225 (5)	0.0008 (4)	0.0149 (4)	0.0011 (4)
C22	0.0292 (5)	0.0172 (4)	0.0235 (5)	−0.0019 (4)	0.0148 (5)	−0.0006 (4)
C23	0.0356 (6)	0.0241 (5)	0.0255 (6)	−0.0017 (4)	0.0168 (5)	0.0017 (4)
C24	0.0425 (8)	0.0246 (5)	0.0265 (5)	−0.0021 (5)	0.0197 (6)	0.0024 (4)
C25	0.0476 (8)	0.0288 (6)	0.0322 (6)	−0.0037 (5)	0.0279 (6)	0.0014 (5)
C26	0.0361 (6)	0.0256 (5)	0.0320 (6)	−0.0019 (5)	0.0225 (5)	−0.0005 (5)
C27	0.0295 (6)	0.0175 (5)	0.0251 (5)	−0.0033 (4)	0.0169 (5)	−0.0016 (4)
C28	0.0267 (5)	0.0191 (4)	0.0260 (5)	−0.0017 (4)	0.0154 (4)	−0.0004 (4)
C29	0.0316 (7)	0.0426 (8)	0.0702 (11)	0.0122 (6)	0.0260 (8)	0.0186 (8)
C30	0.0261 (6)	0.0766 (13)	0.0359 (7)	0.0093 (7)	0.0112 (5)	0.0214 (8)
C31	0.0491 (10)	0.0669 (12)	0.0586 (10)	−0.0142 (9)	0.0367 (9)	0.0017 (9)
C32	0.0690 (14)	0.0621 (13)	0.0694 (13)	−0.0066 (10)	0.0575 (12)	0.0031 (10)

### Geometric parameters (Å, °)

**Table:**

C10—C20A	1.434 (10)	C32—H32A	0.9600
C10—C11	1.5069 (15)	C32—H32B	0.9600
C10—C9	1.5144 (14)	C32—H32C	0.9600
C10—C20	1.5355 (14)	O1A—C1A	1.334 (11)
C10—C9A	1.594 (8)	O1A—C2A	1.410 (10)
C10—C11A	1.631 (9)	O2A—C19A	1.356 (11)
C10—H10A	0.9621	O2A—C18A	1.398 (11)
O1—C1	1.3626 (15)	O3A—C21A	1.342 (10)
O1—C2	1.3774 (14)	O3A—C22A	1.378 (10)
O2—C19	1.3612 (15)	O4A—C1A	1.224 (12)
O2—C18	1.3718 (16)	O5A—C8A	1.320 (9)
O3—C21	1.3489 (13)	O5A—H5AA	0.8200
O3—C22	1.3698 (14)	O6A—C12A	1.292 (10)
O4—C1	1.2270 (14)	O6A—H6AB	0.8200
O5—C8	1.3345 (15)	O7A—C19A	1.231 (11)
O5—H5A	0.8200	O8A—C28A	1.254 (10)
O6—C12	1.3320 (13)	C1A—C9A	1.472 (12)
O6—H6B	0.8200	C2A—C7A	1.378 (10)
O7—C19	1.2235 (16)	C2A—C3A	1.389 (11)
O8—C28	1.2309 (15)	C3A—C4A	1.390 (14)
C1—C9	1.4312 (16)	C3A—H3AA	0.9300
C2—C3	1.3865 (16)	C4A—C5A	1.408 (15)
C2—C7	1.3876 (18)	C4A—C29A	1.518 (14)
C3—C4	1.386 (2)	C5A—C6A	1.416 (14)
C3—H3A	0.9300	C5A—C30A	1.482 (15)
C4—C5	1.411 (2)	C6A—C7A	1.338 (10)
C4—C29	1.514 (2)	C6A—H6AA	0.9300
C5—C6	1.390 (3)	C7A—C8A	1.459 (10)
C5—C30	1.498 (2)	C8A—C9A	1.324 (10)
C6—C7	1.4015 (17)	C11A—C12A	1.327 (10)
C6—H6A	0.9300	C11A—C19A	1.436 (12)
C7—C8	1.4358 (17)	C12A—C13A	1.429 (11)
C8—C9	1.3651 (14)	C13A—C14A	1.383 (12)
C11—C12	1.3688 (16)	C13A—C18A	1.401 (11)
C11—C19	1.4435 (16)	C14A—C15A	1.485 (14)
C12—C13	1.4446 (17)	C14A—H14B	0.9300
C13—C14	1.3945 (19)	C15A—C16A	1.383 (13)
C13—C18	1.3962 (19)	C15A—C31A	1.548 (15)
C14—C15	1.387 (2)	C16A—C17A	1.336 (12)
C14—H14A	0.9300	C16A—C32A	1.507 (14)
C15—C16	1.407 (3)	C17A—C18A	1.406 (12)
C15—C31	1.522 (3)	C17A—H17B	0.9300
C16—C17	1.371 (3)	C20A—C21A	1.344 (12)
C16—C32	1.509 (2)	C20A—C28A	1.432 (11)
C17—C18	1.3870 (18)	C21A—H21B	0.9300
C17—H17A	0.9300	C22A—C27A	1.366 (11)
C20—C21	1.3472 (18)	C22A—C23A	1.406 (12)
C20—C28	1.4644 (16)	C23A—C24A	1.342 (12)
C21—H21A	0.9300	C23A—H23B	0.9300
C22—C27	1.3823 (19)	C24A—C25A	1.330 (12)
C22—C23	1.3995 (18)	C24A—H24B	0.9300
C23—C24	1.3800 (19)	C25A—C26A	1.343 (12)
C23—H23A	0.9300	C25A—H25B	0.9300
C24—C25	1.389 (2)	C26A—C27A	1.390 (11)
C24—H24A	0.9300	C26A—H26B	0.9300
C25—C26	1.3828 (18)	C27A—C28A	1.530 (10)
C25—H25A	0.9300	C29A—H29D	0.9600
C26—C27	1.4042 (18)	C29A—H29E	0.9600
C26—H26A	0.9300	C29A—H29F	0.9600
C27—C28	1.4741 (16)	C30A—H30D	0.9600
C29—H29A	0.9600	C30A—H30E	0.9600
C29—H29B	0.9600	C30A—H30F	0.9600
C29—H29C	0.9600	C31A—H31D	0.9600
C30—H30A	0.9600	C31A—H31E	0.9600
C30—H30B	0.9600	C31A—H31F	0.9600
C30—H30C	0.9600	C32A—H32D	0.9600
C31—H31A	0.9600	C32A—H32E	0.9600
C31—H31B	0.9600	C32A—H32F	0.9600
C31—H31C	0.9600		
			
C20A—C10—C11	134.2 (4)	C8A—O5A—H5AA	109.5
C20A—C10—C9	98.4 (4)	C12A—O6A—H6AB	109.5
C11—C10—C9	112.09 (8)	O4A—C1A—O1A	118.8 (10)
C11—C10—C20	114.64 (9)	O4A—C1A—C9A	124.4 (10)
C9—C10—C20	114.40 (8)	O1A—C1A—C9A	116.5 (9)
C20A—C10—C9A	118.3 (5)	C7A—C2A—C3A	120.7 (8)
C11—C10—C9A	102.4 (3)	C7A—C2A—O1A	123.8 (8)
C20—C10—C9A	136.9 (3)	C3A—C2A—O1A	115.6 (8)
C20A—C10—C11A	117.7 (5)	C2A—C3A—C4A	118.0 (10)
C9—C10—C11A	103.2 (3)	C2A—C3A—H3AA	121.0
C20—C10—C11A	100.9 (3)	C4A—C3A—H3AA	121.0
C9A—C10—C11A	104.4 (4)	C3A—C4A—C5A	121.7 (12)
C20A—C10—H10A	99.2	C3A—C4A—C29A	119.0 (12)
C11—C10—H10A	103.1	C5A—C4A—C29A	119.3 (12)
C9—C10—H10A	107.0	C4A—C5A—C6A	117.4 (12)
C20—C10—H10A	104.3	C4A—C5A—C30A	118.3 (12)
C9A—C10—H10A	87.1	C6A—C5A—C30A	124.2 (13)
C11A—C10—H10A	127.4	C7A—C6A—C5A	120.5 (9)
C1—O1—C2	120.41 (9)	C7A—C6A—H6AA	119.7
C19—O2—C18	121.04 (9)	C5A—C6A—H6AA	119.7
C21—O3—C22	117.95 (10)	C6A—C7A—C2A	121.7 (7)
C8—O5—H5A	109.5	C6A—C7A—C8A	124.3 (8)
C12—O6—H6B	109.5	C2A—C7A—C8A	113.9 (7)
O4—C1—O1	115.57 (10)	O5A—C8A—C9A	125.2 (8)
O4—C1—C9	124.92 (12)	O5A—C8A—C7A	112.2 (7)
O1—C1—C9	119.51 (10)	C9A—C8A—C7A	122.6 (7)
O1—C2—C3	116.57 (11)	C8A—C9A—C1A	121.4 (8)
O1—C2—C7	121.53 (10)	C8A—C9A—C10	124.2 (7)
C3—C2—C7	121.88 (11)	C1A—C9A—C10	114.1 (7)
C4—C3—C2	119.20 (14)	C12A—C11A—C19A	122.1 (8)
C4—C3—H3A	120.4	C12A—C11A—C10	123.4 (7)
C2—C3—H3A	120.4	C19A—C11A—C10	114.4 (7)
C3—C4—C5	120.82 (15)	O6A—C12A—C11A	127.8 (9)
C3—C4—C29	119.16 (17)	O6A—C12A—C13A	111.0 (8)
C5—C4—C29	120.01 (16)	C11A—C12A—C13A	121.2 (8)
C6—C5—C4	118.28 (14)	C14A—C13A—C18A	117.6 (9)
C6—C5—C30	120.05 (18)	C14A—C13A—C12A	127.1 (9)
C4—C5—C30	121.66 (19)	C18A—C13A—C12A	115.1 (8)
C5—C6—C7	121.72 (14)	C13A—C14A—C15A	117.9 (10)
C5—C6—H6A	119.1	C13A—C14A—H14B	121.1
C7—C6—H6A	119.1	C15A—C14A—H14B	121.1
C2—C7—C6	118.09 (12)	C16A—C15A—C14A	120.9 (11)
C2—C7—C8	118.20 (11)	C16A—C15A—C31A	127.6 (13)
C6—C7—C8	123.69 (14)	C14A—C15A—C31A	111.5 (12)
O5—C8—C9	124.82 (11)	C17A—C16A—C15A	120.3 (10)
O5—C8—C7	115.36 (11)	C17A—C16A—C32A	124.7 (11)
C9—C8—C7	119.81 (11)	C15A—C16A—C32A	115.0 (11)
C8—C9—C1	119.81 (11)	C16A—C17A—C18A	119.6 (10)
C8—C9—C10	121.20 (10)	C16A—C17A—H17B	120.2
C1—C9—C10	118.95 (9)	C18A—C17A—H17B	120.2
C12—C11—C19	119.07 (11)	O2A—C18A—C13A	123.7 (8)
C12—C11—C10	125.27 (9)	O2A—C18A—C17A	112.6 (8)
C19—C11—C10	115.44 (10)	C13A—C18A—C17A	123.7 (9)
O6—C12—C11	124.86 (11)	O7A—C19A—O2A	116.3 (9)
O6—C12—C13	114.82 (11)	O7A—C19A—C11A	125.3 (9)
C11—C12—C13	120.27 (11)	O2A—C19A—C11A	118.3 (9)
C14—C13—C18	118.25 (12)	C21A—C20A—C28A	119.5 (9)
C14—C13—C12	124.36 (14)	C21A—C20A—C10	121.8 (8)
C18—C13—C12	117.38 (12)	C28A—C20A—C10	118.1 (7)
C15—C14—C13	120.78 (14)	O3A—C21A—C20A	128.4 (9)
C15—C14—H14A	119.6	O3A—C21A—H21B	115.8
C13—C14—H14A	119.6	C20A—C21A—H21B	115.8
C14—C15—C16	119.57 (16)	C27A—C22A—O3A	120.5 (8)
C14—C15—C31	118.35 (16)	C27A—C22A—C23A	121.4 (8)
C16—C15—C31	122.05 (15)	O3A—C22A—C23A	118.1 (8)
C17—C16—C15	120.19 (16)	C24A—C23A—C22A	117.8 (9)
C17—C16—C32	120.3 (2)	C24A—C23A—H23B	121.1
C15—C16—C32	119.47 (19)	C22A—C23A—H23B	121.1
C16—C17—C18	119.65 (15)	C25A—C24A—C23A	120.6 (9)
C16—C17—H17A	120.2	C25A—C24A—H24B	119.7
C18—C17—H17A	120.2	C23A—C24A—H24B	119.7
O2—C18—C17	116.80 (11)	C24A—C25A—C26A	123.4 (10)
O2—C18—C13	121.68 (11)	C24A—C25A—H25B	118.3
C17—C18—C13	121.49 (13)	C26A—C25A—H25B	118.3
O7—C19—O2	116.02 (10)	C25A—C26A—C27A	118.7 (9)
O7—C19—C11	124.61 (13)	C25A—C26A—H26B	120.7
O2—C19—C11	119.35 (11)	C27A—C26A—H26B	120.7
C21—C20—C28	118.97 (10)	C22A—C27A—C26A	118.1 (8)
C21—C20—C10	121.06 (10)	C22A—C27A—C28A	122.6 (8)
C28—C20—C10	119.56 (11)	C26A—C27A—C28A	119.3 (8)
C20—C21—O3	126.09 (11)	O8A—C28A—C20A	124.8 (8)
C20—C21—H21A	117.0	O8A—C28A—C27A	123.3 (7)
O3—C21—H21A	117.0	C20A—C28A—C27A	111.9 (7)
O3—C22—C27	121.94 (11)	C4A—C29A—H29D	109.5
O3—C22—C23	115.90 (12)	C4A—C29A—H29E	109.5
C27—C22—C23	122.16 (12)	H29D—C29A—H29E	109.5
C24—C23—C22	118.10 (13)	C4A—C29A—H29F	109.5
C24—C23—H23A	121.0	H29D—C29A—H29F	109.5
C22—C23—H23A	121.0	H29E—C29A—H29F	109.5
C23—C24—C25	121.06 (12)	C5A—C30A—H30D	109.5
C23—C24—H24A	119.5	C5A—C30A—H30E	109.5
C25—C24—H24A	119.5	H30D—C30A—H30E	109.5
C26—C25—C24	120.13 (12)	C5A—C30A—H30F	109.5
C26—C25—H25A	119.9	H30D—C30A—H30F	109.5
C24—C25—H25A	119.9	H30E—C30A—H30F	109.5
C25—C26—C27	120.16 (13)	C15A—C31A—H31D	109.5
C25—C26—H26A	119.9	C15A—C31A—H31E	109.5
C27—C26—H26A	119.9	H31D—C31A—H31E	109.5
C22—C27—C26	118.39 (11)	C15A—C31A—H31F	109.5
C22—C27—C28	120.52 (12)	H31D—C31A—H31F	109.5
C26—C27—C28	121.09 (13)	H31E—C31A—H31F	109.5
O8—C28—C20	122.95 (11)	C16A—C32A—H32D	109.5
O8—C28—C27	122.52 (11)	C16A—C32A—H32E	109.5
C20—C28—C27	114.52 (11)	H32D—C32A—H32E	109.5
C1A—O1A—C2A	121.8 (8)	C16A—C32A—H32F	109.5
C19A—O2A—C18A	119.1 (8)	H32D—C32A—H32F	109.5
C21A—O3A—C22A	116.9 (8)	H32E—C32A—H32F	109.5
			
C2—O1—C1—O4	−171.66 (9)	C2A—O1A—C1A—O4A	172.7 (7)
C2—O1—C1—C9	8.03 (15)	C2A—O1A—C1A—C9A	−1.2 (2)
C1—O1—C2—C3	177.31 (10)	C1A—O1A—C2A—C7A	−0.4 (3)
C1—O1—C2—C7	−1.20 (16)	C1A—O1A—C2A—C3A	179.6 (3)
O1—C2—C3—C4	−177.41 (10)	C7A—C2A—C3A—C4A	−0.2 (7)
C7—C2—C3—C4	1.09 (18)	O1A—C2A—C3A—C4A	179.8 (4)
C2—C3—C4—C5	−1.08 (19)	C2A—C3A—C4A—C5A	0.5 (8)
C2—C3—C4—C29	−179.88 (12)	C2A—C3A—C4A—C29A	178.4 (8)
C3—C4—C5—C6	0.3 (2)	C3A—C4A—C5A—C6A	−0.8 (9)
C29—C4—C5—C6	179.14 (14)	C29A—C4A—C5A—C6A	−178.7 (9)
C3—C4—C5—C30	−178.39 (14)	C3A—C4A—C5A—C30A	−179.5 (10)
C29—C4—C5—C30	0.4 (2)	C29A—C4A—C5A—C30A	2.6 (12)
C4—C5—C6—C7	0.4 (2)	C4A—C5A—C6A—C7A	0.9 (8)
C30—C5—C6—C7	179.16 (13)	C30A—C5A—C6A—C7A	179.5 (10)
O1—C2—C7—C6	178.06 (11)	C5A—C6A—C7A—C2A	−0.6 (8)
C3—C2—C7—C6	−0.36 (18)	C5A—C6A—C7A—C8A	179.2 (5)
O1—C2—C7—C8	−3.57 (17)	C3A—C2A—C7A—C6A	0.3 (6)
C3—C2—C7—C8	178.00 (11)	O1A—C2A—C7A—C6A	−179.8 (4)
C5—C6—C7—C2	−0.4 (2)	C3A—C2A—C7A—C8A	−179.6 (4)
C5—C6—C7—C8	−178.66 (13)	O1A—C2A—C7A—C8A	0.4 (5)
C2—C7—C8—O5	−177.72 (11)	C6A—C7A—C8A—O5A	2.7 (8)
C6—C7—C8—O5	0.55 (18)	C2A—C7A—C8A—O5A	−177.5 (6)
C2—C7—C8—C9	1.32 (17)	C6A—C7A—C8A—C9A	−178.4 (5)
C6—C7—C8—C9	179.59 (12)	C2A—C7A—C8A—C9A	1.4 (7)
O5—C8—C9—C1	−175.66 (12)	O5A—C8A—C9A—C1A	175.6 (6)
C7—C8—C9—C1	5.39 (17)	C7A—C8A—C9A—C1A	−3.1 (8)
O5—C8—C9—C10	1.90 (18)	O5A—C8A—C9A—C10	−10.5 (9)
C7—C8—C9—C10	−177.05 (10)	C7A—C8A—C9A—C10	170.8 (5)
O4—C1—C9—C8	169.48 (11)	O4A—C1A—C9A—C8A	−170.6 (8)
O1—C1—C9—C8	−10.18 (16)	O1A—C1A—C9A—C8A	3.0 (6)
O4—C1—C9—C10	−8.13 (17)	O4A—C1A—C9A—C10	15.0 (8)
O1—C1—C9—C10	172.21 (9)	O1A—C1A—C9A—C10	−171.5 (4)
C20A—C10—C9—C8	128.7 (5)	C20A—C10—C9A—C8A	−45.1 (8)
C11—C10—C9—C8	−86.11 (12)	C11—C10—C9A—C8A	113.1 (5)
C20—C10—C9—C8	141.18 (11)	C9—C10—C9A—C8A	−2.5 (4)
C9A—C10—C9—C8	−14.2 (6)	C20—C10—C9A—C8A	−36.2 (7)
C11A—C10—C9—C8	−110.2 (3)	C11A—C10—C9A—C8A	88.0 (6)
C20A—C10—C9—C1	−53.7 (5)	C20A—C10—C9A—C1A	129.2 (6)
C11—C10—C9—C1	91.47 (11)	C11—C10—C9A—C1A	−72.6 (4)
C20—C10—C9—C1	−41.24 (13)	C9—C10—C9A—C1A	171.8 (8)
C9A—C10—C9—C1	163.3 (6)	C20—C10—C9A—C1A	138.1 (4)
C11A—C10—C9—C1	67.4 (3)	C11A—C10—C9A—C1A	−97.7 (5)
C20A—C10—C11—C12	50.1 (7)	C20A—C10—C11A—C12A	−137.7 (9)
C9—C10—C11—C12	−77.94 (13)	C11—C10—C11A—C12A	0.6 (5)
C20—C10—C11—C12	54.65 (13)	C9—C10—C11A—C12A	115.2 (8)
C9A—C10—C11—C12	−102.8 (3)	C20—C10—C11A—C12A	−126.3 (8)
C11A—C10—C11—C12	−5.1 (7)	C9A—C10—C11A—C12A	88.9 (9)
C20A—C10—C11—C19	−135.3 (6)	C20A—C10—C11A—C19A	39.3 (10)
C9—C10—C11—C19	96.63 (11)	C11—C10—C11A—C19A	177.6 (14)
C20—C10—C11—C19	−130.78 (10)	C9—C10—C11A—C19A	−67.8 (8)
C9A—C10—C11—C19	71.8 (3)	C20—C10—C11A—C19A	50.7 (8)
C11A—C10—C11—C19	169.4 (8)	C9A—C10—C11A—C19A	−94.1 (9)
C19—C11—C12—O6	−171.62 (10)	C19A—C11A—C12A—O6A	172.9 (11)
C10—C11—C12—O6	2.77 (17)	C10—C11A—C12A—O6A	−10.3 (16)
C19—C11—C12—C13	11.37 (15)	C19A—C11A—C12A—C13A	−6.7 (16)
C10—C11—C12—C13	−174.24 (10)	C10—C11A—C12A—C13A	170.1 (8)
O6—C12—C13—C14	−1.77 (17)	O6A—C12A—C13A—C14A	5.4 (17)
C11—C12—C13—C14	175.53 (11)	C11A—C12A—C13A—C14A	−174.9 (11)
O6—C12—C13—C18	179.45 (10)	O6A—C12A—C13A—C18A	−178.5 (10)
C11—C12—C13—C18	−3.26 (16)	C11A—C12A—C13A—C18A	1.2 (16)
C18—C13—C14—C15	1.59 (19)	C18A—C13A—C14A—C15A	0.7 (19)
C12—C13—C14—C15	−177.19 (12)	C12A—C13A—C14A—C15A	176.7 (14)
C13—C14—C15—C16	0.9 (2)	C13A—C14A—C15A—C16A	1(2)
C13—C14—C15—C31	−176.94 (14)	C13A—C14A—C15A—C31A	−179.5 (15)
C14—C15—C16—C17	−2.6 (3)	C14A—C15A—C16A—C17A	−2(3)
C31—C15—C16—C17	175.13 (17)	C31A—C15A—C16A—C17A	178.3 (18)
C14—C15—C16—C32	178.38 (17)	C14A—C15A—C16A—C32A	178.1 (15)
C31—C15—C16—C32	−3.9 (3)	C31A—C15A—C16A—C32A	−1(3)
C15—C16—C17—C18	1.8 (2)	C15A—C16A—C17A—C18A	2(2)
C32—C16—C17—C18	−179.22 (16)	C32A—C16A—C17A—C18A	−178.6 (13)
C19—O2—C18—C17	−176.24 (11)	C19A—O2A—C18A—C13A	−5.1 (16)
C19—O2—C18—C13	5.45 (16)	C19A—O2A—C18A—C17A	177.1 (11)
C16—C17—C18—O2	−177.54 (14)	C14A—C13A—C18A—O2A	−178.7 (10)
C16—C17—C18—C13	0.8 (2)	C12A—C13A—C18A—O2A	4.7 (16)
C14—C13—C18—O2	175.78 (11)	C14A—C13A—C18A—C17A	−1.2 (18)
C12—C13—C18—O2	−5.36 (17)	C12A—C13A—C18A—C17A	−177.7 (11)
C14—C13—C18—C17	−2.45 (18)	C16A—C17A—C18A—O2A	177.8 (10)
C12—C13—C18—C17	176.41 (11)	C16A—C17A—C18A—C13A	0.0 (19)
C18—O2—C19—O7	−175.43 (10)	C18A—O2A—C19A—O7A	176.3 (10)
C18—O2—C19—C11	2.96 (15)	C18A—O2A—C19A—C11A	−0.4 (16)
C12—C11—C19—O7	166.87 (11)	C12A—C11A—C19A—O7A	−170.1 (12)
C10—C11—C19—O7	−8.06 (16)	C10—C11A—C19A—O7A	12.9 (17)
C12—C11—C19—O2	−11.37 (16)	C12A—C11A—C19A—O2A	6.3 (17)
C10—C11—C19—O2	173.71 (9)	C10—C11A—C19A—O2A	−170.7 (9)
C20A—C10—C20—C21	−176.1 (14)	C11—C10—C20A—C21A	−166.5 (7)
C11—C10—C20—C21	13.43 (14)	C9—C10—C20A—C21A	−34.0 (11)
C9—C10—C20—C21	144.92 (10)	C20—C10—C20A—C21A	−179 (2)
C9A—C10—C20—C21	160.2 (4)	C9A—C10—C20A—C21A	−16.8 (13)
C11A—C10—C20—C21	34.9 (3)	C11A—C10—C20A—C21A	−143.8 (9)
C20A—C10—C20—C28	−3.5 (14)	C11—C10—C20A—C28A	22.7 (13)
C11—C10—C20—C28	−173.96 (9)	C9—C10—C20A—C28A	155.2 (8)
C9—C10—C20—C28	−42.48 (13)	C20—C10—C20A—C28A	10.5 (7)
C9A—C10—C20—C28	−27.2 (4)	C9A—C10—C20A—C28A	172.3 (7)
C11A—C10—C20—C28	−152.5 (3)	C11A—C10—C20A—C28A	45.3 (11)
C28—C20—C21—O3	0.20 (17)	C22A—O3A—C21A—C20A	1.7 (16)
C10—C20—C21—O3	172.84 (10)	C28A—C20A—C21A—O3A	−3.0 (18)
C22—O3—C21—C20	−0.82 (17)	C10—C20A—C21A—O3A	−173.7 (9)
C21—O3—C22—C27	1.23 (15)	C21A—O3A—C22A—C27A	−0.5 (13)
C21—O3—C22—C23	−178.99 (10)	C21A—O3A—C22A—C23A	179.2 (9)
O3—C22—C23—C24	−179.25 (11)	C27A—C22A—C23A—C24A	−0.2 (15)
C27—C22—C23—C24	0.53 (17)	O3A—C22A—C23A—C24A	−179.9 (9)
C22—C23—C24—C25	−0.91 (19)	C22A—C23A—C24A—C25A	−0.7 (16)
C23—C24—C25—C26	0.6 (2)	C23A—C24A—C25A—C26A	1.6 (18)
C24—C25—C26—C27	0.06 (19)	C24A—C25A—C26A—C27A	−1.7 (17)
O3—C22—C27—C26	179.91 (10)	O3A—C22A—C27A—C26A	179.9 (9)
C23—C22—C27—C26	0.14 (17)	C23A—C22A—C27A—C26A	0.1 (14)
O3—C22—C27—C28	−1.03 (16)	O3A—C22A—C27A—C28A	0.9 (14)
C23—C22—C27—C28	179.20 (11)	C23A—C22A—C27A—C28A	−178.8 (9)
C25—C26—C27—C22	−0.44 (18)	C25A—C26A—C27A—C22A	0.8 (14)
C25—C26—C27—C28	−179.50 (11)	C25A—C26A—C27A—C28A	179.7 (9)
C21—C20—C28—O8	178.56 (11)	C21A—C20A—C28A—O8A	−177.6 (10)
C10—C20—C28—O8	5.80 (16)	C10—C20A—C28A—O8A	−6.6 (15)
C21—C20—C28—C27	0.04 (15)	C21A—C20A—C28A—C27A	2.9 (14)
C10—C20—C28—C27	−172.72 (9)	C10—C20A—C28A—C27A	173.9 (8)
C22—C27—C28—O8	−178.16 (11)	C22A—C27A—C28A—O8A	178.4 (9)
C26—C27—C28—O8	0.88 (18)	C26A—C27A—C28A—O8A	−0.5 (14)
C22—C27—C28—C20	0.37 (15)	C22A—C27A—C28A—C20A	−2.1 (13)
C26—C27—C28—C20	179.41 (10)	C26A—C27A—C28A—C20A	179.0 (9)

### Hydrogen-bond geometry (Å, °)

**Table:**

Cg1, Cg2 and Cg3 are the centroids of the C13–C18, C2–C7 and C2A–C7A benzene rings, respectively.

**Table:**

*D*—H···*A*	*D*—H	H···*A*	*D*···*A*	*D*—H···*A*
O5—H5A···O7	0.82	1.83	2.6464 (19)	174
O6—H6B···O4	0.82	1.80	2.6141 (18)	172
C3—H3A···Cg1^i^	0.93	2.91	3.7691 (16)	155
C32—H32B···Cg2^ii^	0.96	2.70	3.499 (3)	140
C29A—H29D···Cg3^iii^	0.96	2.91	3.591 (15)	128

Symmetry codes: (i) −*x*+1, *y*−1/2, −*z*+3/2; (ii) *x*−1, *y*, *z*; (iii) −*x*+2, −*y*+1, −*z*+2.
